# Improving the prospective prediction of a near-term suicide attempt in veterans at risk for suicide, using a go/no-go task

**DOI:** 10.1017/S0033291722001003

**Published:** 2023-07

**Authors:** Catherine E. Myers, Chintan V. Dave, Michael Callahan, Megan S. Chesin, John G. Keilp, Kevin D. Beck, Lisa A. Brenner, Marianne S. Goodman, Erin A. Hazlett, Alexander B. Niculescu, Lauren St. Hill, Anna Kline, Barbara H. Stanley, Alejandro Interian

**Affiliations:** 1 Research Service, VA New Jersey Health Care System, East Orange, NJ, USA; 2 Department of Pharmacology, Physiology & Neuroscience, New Jersey Medical School, Rutgers, The State University of New Jersey, Newark, NJ, USA; 3 Center for Pharmacoepidemiology and Treatment Science, Institute for Health, Health Care Policy and Aging Research; Rutgers University, New Brunswick, NJ, USA; 4 Mental Health and Behavioral Sciences, VA New Jersey Health Care System, Lyons, NJ, USA; 5 Department of Psychology, William Patterson University, Wayne, NJ, USA; 6 Department of Psychiatry, Columbia University College of Physicians and Surgeons, New York State Psychiatric Institute, New York, NY, USA; 7 VA Rocky Mountain Mental Illness Research Education and Clinical Center, Eastern Colorado Health Care System, Aurora, CO, USA; 8 Departments of Physical Medicine and Rehabilitation, Psychiatry, and Neurology, University of Colorado, Anschutz Medical Campus, Aurora, CO, USA; 9 VISN 2 Mental Illness, Research, Education and Clinical Center (MIRECC), James J. Peters VA Medical Center, Bronx, NY, USA; 10 Department of Psychiatry, Icahn School of Medicine at Mount Sinai, New York, NY, USA; 11 Department of Psychiatry, Indiana University School of Medicine, Indianapolis, IN, USA; 12 Indianapolis Veterans Affairs Medical Center, Indianapolis, IN, USA; 13 Department of Psychiatry, Robert Wood Johnson Medical School, Rutgers, The State University of New Jersey, Piscataway, NJ, USA

**Keywords:** Suicide prediction, impulsivity, response inhibition, Go/No-go, computational model, linear ballistic accumulator

## Abstract

**Background:**

Neurocognitive testing may advance the goal of predicting near-term suicide risk. The current study examined whether performance on a Go/No-go (GNG) task, and computational modeling to extract latent cognitive variables, could enhance prediction of suicide attempts within next 90 days, among individuals at high-risk for suicide.

**Method:**

136 Veterans at high-risk for suicide previously completed a computer-based GNG task requiring rapid responding (Go) to target stimuli, while withholding responses (No-go) to infrequent foil stimuli; behavioral variables included false alarms to foils (failure to inhibit) and missed responses to targets. We conducted a secondary analysis of these data, with outcomes defined as actual suicide attempt (ASA), other suicide-related event (OtherSE) such as interrupted/aborted attempt or preparatory behavior, or neither (noSE), within 90-days after GNG testing, to examine whether GNG variables could improve ASA prediction over standard clinical variables. A computational model (linear ballistic accumulator, LBA) was also applied, to elucidate cognitive mechanisms underlying group differences.

**Results:**

On GNG, increased miss rate selectively predicted ASA, while increased false alarm rate predicted OtherSE (without ASA) within the 90-day follow-up window. In LBA modeling, ASA (but not OtherSE) was associated with decreases in decisional efficiency to targets, suggesting differences in the evidence accumulation process were specifically associated with upcoming ASA.

**Conclusions:**

These findings suggest that GNG may improve prediction of near-term suicide risk, with distinct behavioral patterns in those who will attempt suicide within the next 90 days. Computational modeling suggests qualitative differences in cognition in individuals at near-term risk of suicide attempt.

## Introduction

A priority in suicide risk assessment is the identification not only of individuals who are at risk of dying by suicide, but also the periods when their risk is elevated (for review, Glenn & Nock, 2014). Because intensive clinical and supportive interventions are difficult to apply for long periods of time, a means to identify individuals at near-term risk of suicide can significantly aid suicide prevention efforts by informing which among a population of at-risk individuals can benefit from intensive efforts during an upcoming window of time. While many epidemiological risk factors have been identified that associate with lifetime risk of suicide attempt, these may be distinguished from warning signs or near-term factors that indicate more proximal risk within an upcoming window of days to weeks (Franklin et al., 2016). Illustrating how little is known about proximal risk, a meta-analysis of 50 years of suicide risk factor research reported a mean follow-up period across studies identifying risk factors of 10 years, with only 5% of studies examining a follow-up period of ⩽6 months (Franklin et al., 2016). This is likely due to an inherent methodological challenge: using shorter follow-up periods reduces the likelihood of observing a rare event such as a suicide attempt (Glenn & Nock, 2014). Despite these challenges, detection of warning signs to indicate proximal risk would be of immense clinical value (Gordon, Avenevoli, & Pearson, 2020) and new research in this area is emerging (e.g. Bagge et al., 2022).

Suicidal behavior has been linked to identifiable behavior patterns on a range of neurocognitive tasks (Richard-Devantoy, Berlim, & Jollant, 2014). Such tasks can provide objective markers, and also potentially provide a window into cognitive mechanisms involved with suicidal behavior. For example, reduced ability to inhibit prepotent motor responses has been observed in individuals with a history of suicidal behavior (Mann et al., 2009). One such task, the Go/No-go (GNG) task, requires rapidly responding (‘Go’) to target stimuli but occasionally withholding responses (‘No-go’) to infrequent foil stimuli. Several retrospective cross-sectional studies report increased false alarm rates (i.e., failures to inhibit) in participants with a history of suicide attempt (e.g. Richard-Devantoy et al., 2012; Westheide et al., 2008). In more fine-grained analysis, false alarm rates were higher among patients with a suicide attempt in the prior week compared to those with suicide attempt in the prior year, suggesting reduced response inhibition may be sensitive for near-term suicide attempt (Interian et al., 2020). An important open question is therefore whether cognitive processes may be associated with, and even predict, short-term risk.

As a first step toward this goal, the current study builds on existing research by examining whether GNG performance is prospectively associated with suicide attempt. The study considers a sample of participants at high-risk for suicide enrolled in a randomized clinical trial (RCT). The trial, which showed reductions in suicidal behavior (Interian et al., 2021), observed suicide-related events with enough frequency to meaningfully examine shorter observation windows. Many of these participants also completed GNG at baseline and at several points during a follow-up year. Our primary hypothesis was that GNG performance was predictive of near-term (90-days) suicide attempt, above and beyond standard variables used in suicide risk assessment (i.e., number of previous suicide attempts, level of suicidal ideation).

We also applied a computational model to determine whether observed differences in GNG performance could be understood in terms of altered latent cognitive processes among those with an upcoming suicide attempt. Evidence accumulation models such as the linear ballistic accumulator (LBA) assume that, during speeded decision-making, evidence is gradually accumulated for possible responses, until a winning response is triggered. These models attempt to fit the entire distribution of reaction times (RTs) for correct and incorrect responses, and estimate individual-level latent cognitive parameters, such as response bias, response caution, and decisional efficiency, that may help explain observable behavior. Here, we applied a Bayesian version of the LBA (Brown & Heathcote, 2008; Donkin, Brown, Heathcote, & Wagenmakers, 2011) to the GNG data. Our exploratory hypothesis was that one or more LBA variables would be predictive of near-term suicide attempt, above and beyond the standard suicide risk variables, potentially suggesting specific cognitive processes altered in at-risk individuals entering a period of high risk for suicide attempt.

## Methods

### Participants

This is a secondary analysis of data from 136 Veterans enrolled in a 12-month RCT of Mindfulness-Based Cognitive Behavioral Therapy for Suicide Prevention (MBCT-S) (Interian et al., 2021). Veterans were recruited at VA New Jersey Health Care System (VANJHCS) following an index suicide-related episode, ranging from suicide attempt (SA)to suicidal ideation resulting in acute hospitalization and/or engagement with Veterans Health Administration (VHA) suicide prevention services (Katz, 2012; Stanley & Brown, 2012). These services included suicide safety planning, clinical monitoring and attempts to engage in regular mental health care.

Inclusion criteria for the RCT were both (1) severe suicidal ideation in the prior 30 days and (2) past-year actual, aborted, or interrupted suicide attempt (Posner, Brodsky, Yershova, Buchanan, & Mann, 2014) or placement on the VHA high-risk for suicide list. Full inclusion and exclusion criteria for the RCT are provided in the Supplementary material. As part of the RCT, all participants had access to a full range of standard mental health treatments; the treatment condition also received MBCT-S.

### Overview of procedures

At baseline (T1), participants completed a clinical interview and several questionnaires (Interian et al., 2021; Kline et al., 2016). Suicide behavior counts and worst-point suicidal ideation severity were determined using the Columbia Suicide Severity Rating Scale (C-SSRS) (Posner et al., 2011), using previously-published case classification criteria (Interian et al., 2018). Suicidal ideation severity during the prior week was assessed with the Beck Scale for Suicidal Ideation (SSI) (Beck, Kovacs, & Weissman, 1979).

Participants were followed for one year, with follow-up testing (T2, T3) approximately 3 and 6 months post-T1. These follow-up sessions included updated C-SSRS (covering the period since last session) and SSI (covering prior week).

At each session, participants completed several computer-based tests of neurocognitive processes, including GNG and a color-word Stroop interference task (MacLeod, 1991; Stroop, 1935). In most cases, neurocognitive testing occurred immediately after collection of clinical and self-report data.

### Go-no-go (GNG) task

**Fig. 1.**
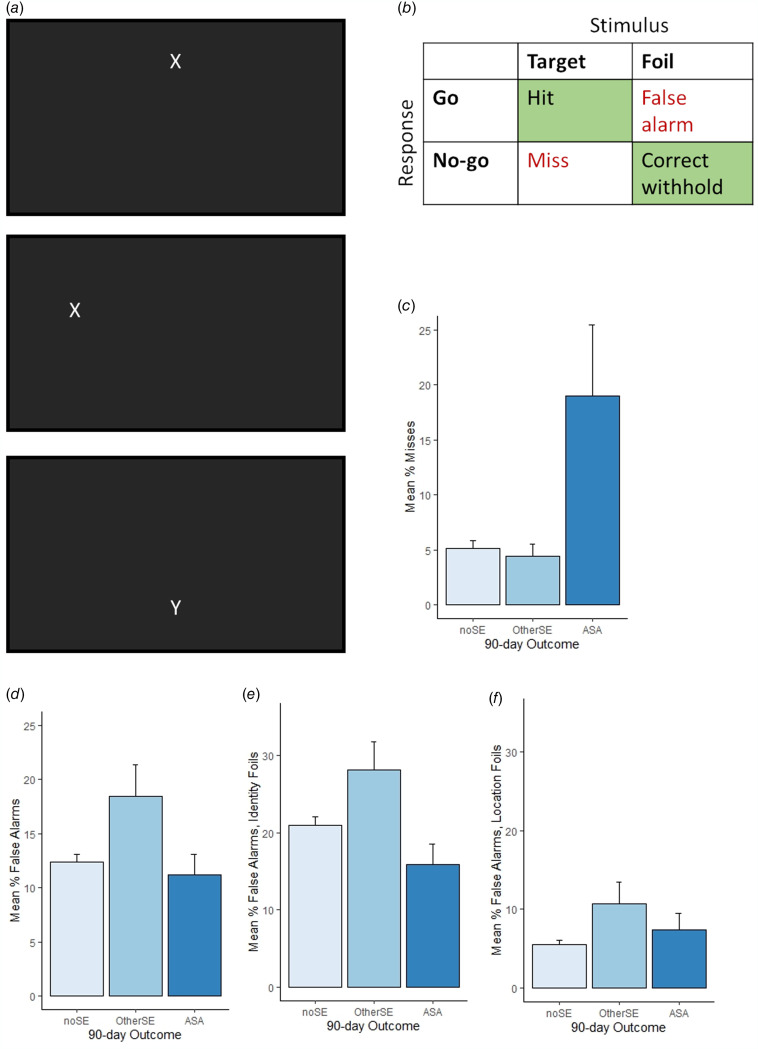
Go/No-go task and results, across all *n* = 284 GNG datafiles; note participants who completed more than one testing session are represented more than once. (*a*) Participants were instructed to respond (Go) to rapidly-presented target stimuli (X in upper half of screen) but withhold response (No-go) to infrequent identity foils (Y in upper half of screen) and location foils (X or Y in lower half of screen). Top and middle show example screenshots on target trials; bottom shows example location foil trial. (*b*) Go responses to targets were scored as Hits and No-go responses as misses (aka omission errors). No-go responses to foils were scored as correct withholds, and Go responses as false alarms (aka commission errors). (*c*) Misses were highest when 90-day follow-up included an actual suicide attempt (ASA group, *n* = 18), compared to outcomes including other suicide-related event excluding ASA (OtherSE group, *n* = 29) or no suicide-related event within follow-up window (noSE, *n* = 237). (*d*) In contrast, false alarms were higher in the OtherSE group compared to the ASA or noSE groups. (*e*) False alarm rates to identity foils (Y in correct location) and (*f*) false alarm rates to location foils (X or Y in incorrect location). Error bars show SEM.

The GNG task was previously described (Interian et al., 2020; Keilp et al., 2014a; Moore et al., 2019) and is adapted from the original bimodal matching GNG task described in Keilp, Sackeim, and Mann (2005). On each trial, an X or Y appeared on the screen (Fig. 1*a*). Participants were instructed to press a key (‘Go response’) when X appeared in one of three locations in the top half of the screen area (target), but to withhold keypresses (‘No-go response’) when Y appeared in one of the upper locations (identity foil), or when either X or Y appeared in one of three locations in the bottom half of the screen (location foil). Stimuli appeared for 300 ms followed by 1200 ms blank screen (total trial length 1.5 s). The task included 225 trials, including 144 targets and 81 foils (36 identity foils; 45 location foils including 36 X and 9 Y); in one instance, only the first 85 trials were recorded due to computer failure.

Following prior studies with GNG tasks (e.g. Gomez, Ratcliff, & Perea, 2007; Huang-Pollock et al., 2020; Ratcliff, Huang-Pollock, & McKoon, 2018; Weigard & Huang-Pollock, 2017), we discarded responses with RT < 200 ms as anticipatory responses, and discarded responses with RT > 1s as likely reflecting inattention or other interfering cognitive processes (Lerche & Voss, 2019; Ratcliff, 1993). A mean of 0.96 short-RT trials (s.d. 4.3) and 0.80 long-RT trials (s.d. 1.76) per GNG datafile were dropped.

Key behavioral variables (Fig. 1*b*) were percent misses (aka omission errors) and percent false alarms (aka commission errors).

### Stroop task

The color-word Stroop task was previously described (Interian et al., 2020; Keilp et al., 2014a; Moore et al., 2019). On each trial, participants saw a word (RED, GREEN, or BLUE) and pressed a key to indicate the font color (red, blue, or green). The task included 52 congruent trials (e.g., word RED printed in red font) and 58 incongruent trials (e.g., word RED in green font). Words remained present on screen until the correct response was made, with intertrial intervals of 50 ms.

A d-score for interference was calculated as the difference between mean RT for incongruent *v.* congruent trials, divided by mean RT for congruent trials; higher (positive) d-scores indicated greater interference and thus poorer attentional control.

### LBA modeling

**Fig. 2.**
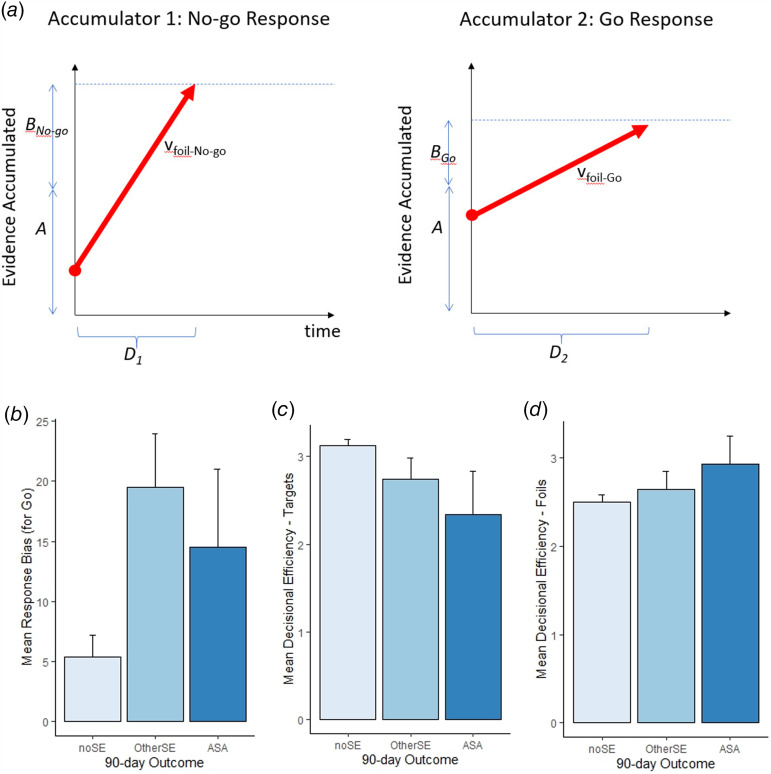
The linear ballistic accumulator model (LBA) adapted to apply to Go/No-go task. (*a*) Schematic of the LBA model, showing one evidence accumulator for each response (here, No-go and Go); at the start of each trial, a starting point for each accumulator is drawn from the uniform distribution U [0…A]; evidence accumulation in each accumulator then follows a trajectory (red lines) with slope drawn from a normal distribution with mean *v* (where *v* may be different in each accumulator and for each stimulus type). The first accumulator to reach a threshold *A* + *B* (dashed line) ‘wins’ and the corresponding response is triggered. In the example shown here, boundary offset *B_No−go_ > B_Go_*, creating a relative bias in favor of Go responses (less distance to travel to reach threshold in the Go accumulator); however, the mean slope *v* on foil trials is greater in the No-go than Go accumulator, meaning that evidence accumulation proceeds more swiftly in the No-go accumulator, favoring the correct (No-go) response. Mean slope *v* on target trials (not shown) is typically steeper in the Go than the No-go accumulator, favoring the correct (Go) response. Total reaction time (RT) on this trial is the time for the winning accumulator to reach threshold plus non-decision time (*t0*) representing time to encode the stimulus and execute the response. Variability in RT and response across trials is provided by trial-to-trial variability in starting point and in slope. Values of eight free parameters (*t0, A, B_No-go_, B_Go_,* and *v* for each combination of stimulus and response) are imputed for each datafile such that the resulting LBA model best predicts the observed RT distributions. (*b*) Response bias for Go responses, defined as 100*(*B_No–go_ – B_Go_*) is greatest in the OtherSE group, consistent with this group's high rate of false alarms. (*c*, *d*) Decisional efficiency for targets and foils are defined as the difference in *v* between correct and incorrect responses to that type of stimulus, where larger (positive) values indicate more efficiency in deciding to execute the correct response; here, the ASA group has lowest decisional efficiency for targets, consistent with this group's relatively high miss rate, and the highest decisional efficiency for foils. Error bars show SEM.

A Bayesian version of the LBA adapted for the GNG task (Fig. 2*a*) was fit to each GNG datafile, using the Dynamic Models of Choice (DMC) package *v.* 190819 (Heathcote et al., 2019) and base R functions (R Core Team, 2017) to estimate posterior distributions for eight latent cognitive variables: non-decision time (*t0*), starting point variability (*A*), boundary offsets (*B_No−go_* and *B_Go_* for No-go and Go accumulators, respectively), and mean slope parameters (v) for each combination of stimulus type (foil *v.* target) and accumulator (Go *v.* No-go). Full details of LBA model building and testing appear in the Supplementary material.

The medians of posterior distributions for each datafile were used as point estimates (Zhang et al., 2016). Following Karalunas, Weigard, and Alperin (2020), after estimating posteriors for each GNG datafile, a measure of response bias for Go responses was calculated as 100*(*B_No_*_−*go*_*-B_Go_*), where values > 0 indicate greater boundary offset for the No-go than Go accumulator (easier to reach threshold for Go responses). Decisional efficiency for targets and foils was calculated as *v_target-Go_–v_target-No-go_ and v_foil-No-go_–v_foil-Go_*, respectively, where larger (positive) values indicate more efficiency in deciding to execute the correct response for that trial type. These three metrics (response bias for Go responses, decisional efficiency for targets, and decisional efficiency for foils) constituted our primary results from the LBA analysis.

### Prospective (90-day) outcome evaluation

For each GNG datafile, we categorized the outcome into one of three mutually exclusive categories: (1) ‘ASA’ if the participant had 1+ actual suicide attempt during the 90 days subsequent to GNG testing; (2) ‘OtherSE’ if the participant had no ASA during this window but at least one other suicide-related event (SE), including interrupted/aborted suicide attempt, preparatory behavior (e.g., writing a note, assembling a method) or suicide-related hospital admission (e.g., emergency department visit or acute psychiatry admission related to suicidal ideation); or (3) ‘noSE’ if the participant had neither ASA nor other SE within the 90-day follow-up window.

ASA and other suicide behaviors were determined from clinician-administered C-SSRS at each available timepoint; medical chart review was used to capture SI-related hospital admissions. In one case, chart review also identified an ASA that occurred after a patient had been lost to contact. In two cases, participants had two GNG sessions occurring within <90 days, and the same ASA fell within the follow-up window for both sessions.

Of the 310 available GNG datafiles, four were dropped because they could not be associated with an outcome due to censoring (death from natural causes, study withdrawal, or study end within <90 days) and an additional 22 (~7%) were dropped due to apparent noncompliance or failure to understand task instructions (participant never made any ‘Go’ responses, and may have been pressing the wrong keyboard key, *n* = 10; participant made 90–100% errors to (only) one type of foil and likely misunderstood the requirement to inhibit responding to both types of foil, *n* = 12). There were no obvious differences in demographics, clinical profile or outcome distribution among these dropped files compared to the remaining 284 GNG files (results not shown).

### Statistical analysis

The dependent variable for all analyses was outcome category: ASA, OtherSE, or noSE (reference category). Generalized estimating equations (GEEs) were used to test the effects of predictors on this multinomial dependent variable. Given that the same patient could contribute multiple data points (maximum of 3 observations per subject), we accounted for within-subject clustering. We also controlled for the effects of the testing session (T1, T2, T3) by modeling it as a categorical factor. Results were reported as odds ratios (OR) with 95% confidence interval (CI); threshold for significance was set at 0.05.

GEE models were estimated using SAS Enterprise (version 7.18, SAS Institute Inc, Cary, NC). First, in a simple model of GNG behavior across outcome groups, we used separate GEEs to model the dependent variable against GNG percent misses (failure to respond Go to target) and GNG percent false alarms (failure to withhold response to foil), adjusting only for testing session number. Second, to test our hypothesis regarding predictive value of behavioral measures, we used a GEE to evaluate the incremental utility of GNG behavioral scores in predicting the response variable, over standard suicide risk variables (number of lifetime ASAs, SSI at time of testing), as well as other pertinent covariates, such as testing session number, age, gender, lifetime history of traumatic brain injury (TBI), receipt of study treatment during the RCT to account for treatment effects on suicide outcomes, and an index of executive attention (Stroop *d*-score).

To explore the LBA variables, the same methods were used, except using LBA estimates of response bias and decisional efficiency as predictors.

## Results

### Sample characteristics

Table 1 summarizes demographic and clinical information for the 136 Veterans. Thirteen (9.6%) participants had 1+ actual suicide attempt (ASA) during the one-year follow-up period, while 25 (18.4%) had no ASA but 1+ OtherSE. After data cleansing, *n* = 284 GNG datafiles obtained from 130 unique participants were analyzed.

**Table 1.** Demographic and clinical information at baseline testing (T1)

	Unique subjects (*n* = 136)	
	*M*	s.d.
	46	13.73
Age (year)	*N*	%
**Gender**		
Female	17	12.50%
Male	119	87.50%
**Race/ethnicity**		
White	60	44.12%
Black	39	28.68%
Asian, American Indian, Other	8	5.88%
Latino	29	21.32%
**Education**		
High school or Graduate Equivalency Degree (GED) or less	46	33.82%
Some college but no degree	62	45.59%
Graduation from 4-year college or higher	28	20.59%
**Employment status**		
Employed	34	25.00%
Unemployed	97	71.32%
Sheltered workshop (e.g. clinical work therapy program)	1	0.74%
Student	3	2.21%
**Marital status**		
Married or living as married	36	26.47%
Never married	35	25.74%
Separated or divorced	60	44.12%
Widowed	4	2.94%
**Psychiatric/Neurological features**		
Head Injury/Traumatic Brain Injury (TBI)	78	57.35%
Post-traumatic stress disorder (PTSD)	82	60.29%
Psychosis	7	5.15%
Social Phobia	5	3.68%
Obsessive-compulsive disorder	4	2.94%
Generalized anxiety disorder	10	7.35%
Major depressive disorder	95	69.85%
Bipolar	17	12.50%
Any substance use in past 30 days	59	43.38%
Binge drinking episode in past 30 days	51	37.50%
**Non-suicidal self-injurious behavior (NSSI) events**		
None	71	52.21%
One or more	65	47.79%
**Suicidal ideation (SI) with some intent**		
CSSR-S ideation severity ⩾4	122	89.71%
**Lifetime actual suicide attempts (prior to study enrollment)**		
None	22	16.18%
One	37	27.21%
Multiple (2 or more)	77	56.62%

GNG datafiles were classified into outcome groups based on the 90-day window following GNG testing, resulting in 18 datafiles classed as ASA (2 from female participants, 11.1%), including one case with two ASAs in the 90-day window. Another 29 were classed as OtherSE (5 from female participants, 17.2%); of these, 9 cases involved SI-related hospital admissions without suicide-related behavior, 7 involved aborted/interrupted attempts, and the remaining 13 cases involved preparatory behavior. The remaining 237 were classed noSE (29 from female participants, 12.2%). Detailed GNG results for each outcome group are summarized in the online Supplementary Table.

### GNG task performance and prediction of near-term suicide outcomes

Compared to the noSE reference group, misses were higher in the ASA group [Fig. 1*c*; OR  1.05, (1.02–1.08), *p* < 0.001], but not in the OtherSE group [OR 0.99, (0.96–1.03), *p* = 0.713]. Conversely, false alarm rates were higher in the OtherSE group [Fig. 1*d*; OR 1.04, (1.01–1.07), *p* = 0.022], but not in the ASA group [OR 0.99, (0.94–1.04), *p* = 0.632]. Figure 1*e* and *f* show false alarms for the two subtypes of foil separately, illustrating that the OtherSE group made more false alarms on both the relatively easy location foils and the more difficult identity foils.

To relate ORs to observed GNG differences, we exponentiated raw beta coefficients multiplied by the observed group mean differences. Thus, while every unit increase in misses corresponded to a 5% increase in odds of ASA, the observed mean difference in misses (14%) corresponded to OR 1.92, or a 92% increase in odds of ASA within the next 90 days. Similarly, while every unit increase in false alarm rate corresponded to a 4% increase in odds of OtherSE, the observed mean difference in false alarms (6%) corresponded to OR 1.25, or a 25% increase in odds of OtherSE (excluding ASA) within the next 90 days.

Our primary hypothesis was that GNG variables could predict upcoming ASA, above and beyond the contributions of other standard suicide risk variables. Table 2 summarizes results of the GEE using GNG variables as predictors, adjusting for suicide risk variables and other pertinent covariates. As expected, suicidal ideation at the time of testing increased the odds of an upcoming ASA while receipt of study treatment in the RCT decreased the odds of ASA. Consistent with our hypothesis, increased rate of GNG misses increased the odds of ASA, independently of the other covariates. Increased rate of GNG false alarms was associated with increased odds of upcoming OtherSE (excluding ASA), independently of the other covariates.

**Table 2.** Predicting suicide-related behavior (actual suicide attempt or other suicidal event excluding ASA) within 90 days based on GNG behavioral variables: results from GEE, with session as repeated-measure, adjusted by key suicide-related covariates

	Actual suicide attempt (ASA)			Other suicidal event (excluding ASA)		
	OR	95% CI	*p* Value	OR	95% CI	*p* Value
GNG: % misses	**1.06**	**1.04–1.09**	**<0.001**	0.98	0.94–1.02	0.37
GNG: % false alarms	0.96	0.91–1.01	0.13	**1.05**	**1.01–1.08**	**<0.01**
Session = T2 (reference = T1)	3.05	0.55–16.97	0.20	0.40	0.11–1.53	0.18
Session = T3 (reference = T1)	0.13	0.02–0.76	0.02	0.61	0.17–2.17	0.45
# Prior ASA (lifetime)	1.16	0.99–1.36	0.06	1.09	0.94–1.25	0.25
Age in years at T1	1.03	0.96–1.10	0.42	0.99	0.96–1.03	0.65
Female gender (reference = male)	0.80	0.10–6.28	0.83	1.61	0.51–5.05	0.42
Lifetime TBI (reference = none)	0.66	0.10–4.21	0.66	1.01	0.36–2.85	0.99
RCT group (reference = MBCT-S)	**14.43**	**1.75–119.14**	**0.01**	1.54	0.56–4.24	0.40
SSI at time of GNG testing	**1.13**	**1.04–1.24**	**<0.01**	1.01	0.96–1.06	0.65
Attentional Control at T1 (Stroop *d*-score)	2.91	0.03–337.98	0.66	0.27	0.00–16.87	0.54

GNG, Go/No-go task; RCT, randomized clinical trial for Mindfulness-Based Cognitive Therapy for Suicide (MBCT-S); SSI, Scale for Suicidal Ideation score.

*Note*. Bold indicates predictors for which *p* < 0.05 for either the Actual Suicide Attempt (ASA) or Other Suicidal Event outcome.

### Latent parameters from evidence accumulation model

Of the 284 GNG datafiles analyzed above, the LBA could not be applied to 22 datafiles containing <2 false alarms (so RT variance could not be calculated). The LBA model was run on the remaining 262 datafiles (17 ASA, 28 OtherSE, 217 noSE).

Posterior parameter estimates are shown along with other detailed results in the online Supplementary Table. As shown in Fig. 2*b*, the OtherSE group had a stronger response bias for Go responding than the noSE reference group [OR 1.02 (1.01–1.04), *p* = 0.009], while the ASA group did not [OR 1.01 (0.99–1.03), *p* = 0.20]. The pattern of stronger response bias for Go in the OtherSE group is consistent with their high rate of false alarms on the GNG task.

The ASA group had lower decisional efficiency for targets [Fig. 2*c*; OR 0.63 (0.46–0.87), *p* = 0.004], while the OtherSE group did not [OR 0.80 (0.57–1.11), *p* = 0.18]. This relative difficulty of the ASA group in accumulating evidence towards a correct response to targets may explain their high rates of misses on the GNG task. Neither the OtherSE nor ASA group differed from the noSE group in decisional efficiency for foils [Fig. 2*d*; OtherSE OR  1.13 (0.80–1.59), *p* = 0.48; ASA OR 1.40 (0.81–2.43), *p* = 0.23].

To relate ORs to observed parameter differences, the observed differences between ASA and noSE on decisional efficiency for targets (−0.79) increased the odds of ASA by 43%. The observed 14-point difference between OtherSE and noSE on response bias increased the odds of OtherSE by 34%.

To test our exploratory hypothesis, Table 3 summarizes results of the GEE using LBA parameters as predictors, adjusting for standard suicide risk variables and other covariates. Response bias (favoring Go responding) was strongly related to upcoming OtherSE, adjusting for the covariates; however, both decisional efficiency for targets and decisional efficiency for foils were significantly related to ASA within 90 days, after adjusting for the covariates: Every unit *increase* in decisional efficiency for foils more than doubled the odds of an ASA; while every unit *decrease* in decisional efficiency for targets increased the odds of an ASA.

**Table 3.** Predicting suicide-related behavior within 90 days based on LBA variables: results from GEE, with session as repeated-measure, adjusted by key suicide-related covariates

	Actual suicide attempt (ASA)			Other suicidal event (excluding ASA)		
	OR	95% CI	*p* Value	OR	95% CI	*p* Value
LBA: Response bias for Go	0.99	0.96–1.01	0.34	**1.03**	**1.00–1.06**	**0.03**
LBA: Decisional efficiency for targets	**0.53**	**0.36–0.79**	**<0.01**	1.06	0.70–1.62	0.77
LBA: Decisional efficiency for foils	**2.46**	**1.26–4.80**	**0.01**	0.67	0.34–1.32	0.25
Session = T2 (T1 reference)	2.63	0.65–10.67	0.18	0.42	0.11–1.56	0.19
Session = T3 (T1 reference)	0.08	0.01–0.53	0.01	0.63	0.19–2.17	0.47
# Prior ASA (lifetime)	1.22	1.03–1.44	0.02	1.10	0.95–1.27	0.21
Age in years at T1	1.04	0.97–1.12	0.27	0.99	0.96–1.03	0.59
Female gender (reference = male)	0.60	0.06–6.08	0.67	1.52	0.41–5.56	0.53
Lifetime TBI (reference = none)	0.66	0.08–5.57	0.70	1.00	0.36–2.78	1.00
RCT group (reference MBCT-S)	**11.78**	**1.12–124.44**	**0.04**	1.46	0.54–3.96	0.45
SSI at time of GNG testing	**1.13**	**1.04–1.23**	**0.01**	1.01	0.95–1.06	0.84
Attentional Control at T1 (Stroop d-score)	4.25	0.08–227.74	0.48	0.35	0.01–11.11	0.55

LBA, Linear ballistic accumulator model; Response bias for Go values >0 indicate bias for Go over No-go responses; Decisional efficiency for targets/foils >0 indicate faster evidence accumulation for correct than incorrect responses to targets and foils, respectively; RCT, randomized clinical trial for Mindfulness-Based Cognitive Therapy for Suicide (MBCT-S); SSI, Scale for Suicidal Ideation score.

*Note*. Bold indicates predictors for which *p* < 0.05 for either the Actual Suicide Attempt (ASA) or Other Suicidal Event outcome.

## Discussion

A priority for suicide prevention is identifying which among a set of individuals considered at high-risk for suicide are most likely to attempt suicide within a short-term window, so that clinical resources can be appropriately targeted. Several prior studies have associated GNG performance with prior suicide attempt (Interian et al., 2020; Richard-Devantoy et al., 2012; Westheide et al., 2008); the current study investigated whether GNG performance could be used to prospectively predict future ASAs.

In our sample of high-risk Veterans, increased false alarms were predictive of an upcoming SE excluding ASA. This is consistent with our own prior analysis showing a relationship between recent (prior) ASA and false alarms at baseline in this dataset (Interian et al., 2020) and other studies associating prior SE with decreased response inhibition (Richard-Devantoy et al., 2012; Westheide et al., 2008). LBA modeling suggested that high false alarm rate in the OtherSE group might reflect a general response bias favoring Go over No-go responses.

However, it was GNG misses, i.e., failures to respond to rapidly-presented targets, that strongly and selectively associated with upcoming ASAs, and that distinguished the ASA group from both the noSE and OtherSE groups. Other prior studies have similarly reported increased miss rates as an important indicator of suicidality (Harfmann, Rhyner, & Ingram, 2019; Westheide et al., 2008; Wright, Lipszyc, Dupuis, Thayapararajah, & Schachar, 2014). The significant association of misses with ASA, after accounting for other variables, also held in a supplemental analysis where the GEE was run with binary outcome coding ASA *v.* all other outcomes (results not shown).

Increased miss rates in the ASA group could reflect several cognitive processes. First, it could reflect a general impairment in attention; however, this group did not show evidence of general attention/concentration deficits as indexed by Stroop task d-scores. Increased miss rate could also reflect a general psychomotor slowing which would reduce the likelihood of completing a Go response before the end of the 1.5-s trial period; however, the ASA group showed no slowing of behavioral RT, compared to the other groups (online Supplementary Table).

To explore specific cognitive processes that could underlie an increased miss rate in the ASA group, we turned to computational modeling, which provides a way to examine and quantify latent processes that could govern individual- and group-level differences in performance, but that are not directly evident from analysis of behavioral indices alone. The model results suggested no evidence of general slowing of stimulus encoding and motor response time (*t0*) nor of a general response bias favoring No-go in the ASA group. Rather, the behavior of those with upcoming ASA was consistent with reduced decisional efficiency for targets (Fig. 2*d*). Here, the reduced decisional efficiency for targets in the ASA (but not OtherSE or noSE) group suggests qualitative, not merely quantitative, differences in cognitive processes in a subset of at-risk individuals who will soon attempt suicide.

A key strength of computational modeling is to propose formal, mechanistic and biologically plausible mechanisms underlying observable behaviors (Millner, Robinaugh, & Nock, 2020). In electrophysiology studies, decisional efficiency has been linked with the build-up rate of the P3b component of the P300 event-related potential (Kelly & O'Connell, 2013), and appears to originate from temporal-parietal activity associated with stimulus detection and attention (Polich, 2007). Interestingly, reduced decisional efficiency is emerging as a neurocognitive risk factor in a range of psychopathologies (for review, see Weigard & Sripada, 2021). A few studies have suggested that decisional efficiency can be increased by catecholamine agonists that modulate brain signal-to-noise ratios (Peters, Vega, Weinstein, Mitchell, & Kayser, 2020; Weigard, Heathcote, & Sripada, 2019), which may be particularly relevant given that brain serotonergic systems have been associated with suicidality (Oquendo et al., 2014; van Heeringen & Mann, 2014). This raises the intriguing possibility of therapeutic intervention to modify brain substrates underlying reduced decisional efficiency, which might in turn help to remediate dysfunctional cognitive and executive processes underlying suicidality. This also illustrates a key utility of computational modeling, which is to identify cognitive phenotypes that can help bridge between brain circuits and clinical behavior.

Further studies are obviously indicated to further explore these ideas, including replication of the current results, before any clinical recommendations can be made. Nevertheless, the strength and specificity of the relationship between misses and ASAs in the current study suggest that GNG misses may be a proximal warning sign of suicide risk, as well as an indicator providing some insight into cognitive processes that may change as high-risk individuals enter a period of acute risk for suicide. If the current results were appropriately replicated/validated, the GNG might represent a fairly short, computer-based screening tool that could be administered in an acute care setting or delivered via Internet or mobile device for more routine follow-up; positive screens (e.g., an increase in an individual's GNG miss rate) could trigger specific interventions including increased clinical encounters, more intensive safety planning, or increased monitoring efforts during periods of elevated risk (Stanley et al., 2018).

The fact that decreased response inhibition (increased false alarm rate) was associated in our study with OtherSE, but not with upcoming ASA, may at first appear at odds with the idea that decreased inhibition might mediate the transition from suicidal thoughts to suicide attempts (i.e., from ideation to action). One possible interpretation is that different neurocognitive deficits may be more prominent during different phases of the transition from suicidal ideation to suicide attempt; for example, decision-making difficulties may lead to a downward spiral of mood and function, while impulsivity may be associated when individuals take action to escape the pain. Another possibility is that there may be different deficits at play with different subpopulations who exhibit different types of attempts. This would be consistent with recent suggestions that cognitive impulsivity (e.g., reduced ability to delay gratification) is decreased in individuals with high-lethality suicide attempts (Anestis, Soberay, Gutierrez, Hernández, & Joiner, 2014; Dombrovski et al., 2011; Keilp et al., 2014b). These, together with the current results, suggest that some attempts may be characterized by decisional difficulties and less reactive responding, while other attempts may be characterized by greater reactivity and impulsivity. The emerging field of ecological momentary assessment may shed light on these questions, by allowing short cognitive tasks to be administered in a more naturalistic setting, potentially allowing the detection of more rapid fluctuations in cognitive processes closer to the time of an upcoming ASA (e.g. Le et al., 2021).

It is also useful to compare rates of GNG errors in our high-risk group against a sample of putatively healthy control participants. For example, Hoffman et al. (2022) administered this same GNG task to a sample of over 38 000 Army Soldiers (17% females; mean age 20.97 years), of whom the vast majority (98%) reported no lifetime history of suicide attempt. This study reported a mean of ~10% false alarms, quite similar to the overall rate in the current study (Fig. 1*c* and online Supplementary Table); a follow-up cohort (including 3.4% with ‘new-onset’ suicide attempt during the 3–7 year follow-up period), again reported ~12% false alarms. Hoffman et al. reported that GNG false alarms was a significant predictor of retrospective history of lifetime suicide attempts, but not a significant predictor in a prospective model predicting emergence of new-onset ASA at follow-up. This seems to lend credence to the finding in the current study that reduced inhibition, as indexed by GNG false alarms, is not a significant prospective predictor, at least when measurements are made weeks or months before the attempt. Again, assessments made more frequently, and/or closer to the time of an upcoming ASA, might detect more rapid fluctuations in these cognitive processes, and thus be more successful prospective predictors.

A key limitation of our study is the low incidence of outcome events (particularly ASAs), even within our high-risk sample. Indeed, the low frequency of attempts, even among high-risk individuals, has been a prediction challenge clinically, but also for research studies. Other limitations of the current study include underrepresentation of females among our Veteran participants which may have masked gender differences, and lack of information on psychotropic medication that could have influenced behavior. Also, about 7% of GNG datafiles were excluded due to apparent subject non-compliance or failure to understand task instructions, and a further ~10% of GNG datafiles could not be subjected to LBA modeling due to too few false alarms, suggesting some participants may have been trading speed for accuracy. It would be interesting to see if the current pattern of results were replicated with a simpler GNG task, putatively requiring less cognitive load than the current task which involved multiple types of foil. It is also important that all participants were enrolled in a treatment trial, which may have modified their behavior across time. Indeed, fewer participants in the MBCT-S treatment group had ASAs during follow-up than those in the control group (Interian et al., 2021). Nevertheless, current results remained significant after adjusting for treatment group effects in the multivariate models. In fact, any study enrolling at-risk participants is likely to be complicated by the ethical necessity for interventions related to suicide prevention.

Despite these limitations, our finding of increased miss rates associated with upcoming suicide attempt suggests there may overt behavioral profiles that can be detected in advance of an upcoming ASA, and also points to the potential utility of neurocognitive deficits to provide objective measures to complement existing clinically-assessed warning signs.

## Supplementary material

For supplementary material accompanying this paper visit https://doi.org/10.1017/S0033291722001003. click here to view supplementary material

## References

[ref1] Anestis, M. D., Soberay, K. A., Gutierrez, P. M., Hernández, T. D., & Joiner, T. E. (2014). Reconsidering the link between impulsivity and suicidal behavior. Personality and Social Psychology Review, 18, 366–386. 24969696 10.1177/1088868314535988

[ref2] Bagge, C. L., Littlefield, A. K., Wiegand, T. J., Hawkins, E., Trim, R. S., Schumacher, J. A., … Conner, K. R. (2022). A controlled examination of acute warning signs for suicide attempts among hospitalized patients. Psychological Medicine, 2022 Jan 25, 1-9. Advance online publication. doi: 10.1017/S0033291721004712. PMC10235647 35074021

[ref3] Beck, A. T., Kovacs, M., & Weissman, A. (1979). Assessment of suicidal intention: The scale for suicide ideation. Journal of Consulting and Clinical Psychology, 47(2), 343–352. 469082 10.1037//0022-006x.47.2.343

[ref4] Brown, S. D., & Heathcote, A. (2008). The simplest complete model of choice response time. Cognitive Psychology, 57(3), 153–178. 18243170 10.1016/j.cogpsych.2007.12.002

[ref5] Dombrovski, A. Y., Szanto, K., Siegle, G. J., Wallace, M. L., Forman, S. D., Sahakian, B., … Clark, L. (2011). Lethal forethought: Delayed reward discounting differentiates high- and low-lethality suicide attempts in old age. Biological Psychiatry, 70(2), 138–144. 21329911 10.1016/j.biopsych.2010.12.025 PMC3125431

[ref6] Donkin, C., Brown, S., Heathcote, A., & Wagenmakers, E.-J. (2011). Diffusion versus linear ballistic accumulation: Different models but the same conclusions about psychological processes? Psychonomic Bulletin and Review, 18, 61–69. 21327360 10.3758/s13423-010-0022-4 PMC3042112

[ref7] Franklin, J. C., Ribeiro, J. D., Fox, K. R., Bentley, K. H., Kleiman, E. M., Huang, X., … Nock, M. K. (2016). Risk factors for suicidal thoughts and behaviors: A meta-analysis of 50 years of research. Psychological Bulletin, 143(2), 187–232. 27841450 10.1037/bul0000084

[ref8] Glenn, C. R., & Nock, M. K. (2014). Improving the short-term prediction of suicidal behavior. American Journal of Preventive Medicine, 47(3), S176–S180. 25145736 10.1016/j.amepre.2014.06.004 PMC5258198

[ref9] Gomez, P., Ratcliff, R., & Perea, M. (2007). A model of the go/no-go lexical decision task. Journal of Experimental Psychology: General, 136, 389–413. 17696690 10.1037/0096-3445.136.3.389 PMC2701630

[ref10] Gordon, J. A., Avenevoli, S., & Pearson, J. L. (2020). Suicide prevention research priorities in health care. JAMA Psychiatry, 77(99), 885–886. doi: 10.1001/jamapsychiatry.2020.1042. 32432690

[ref11] Harfmann, E. J., Rhyner, K. T., & Ingram, R. E. (2019). Cognitive inhibition and attentional biases in the affective go/no-go performance of depressed, suicidal populations. Journal of Affective Disorders, 256, 228–233. 31200162 10.1016/j.jad.2019.05.022

[ref12] Heathcote, A., Lin, Y.-S., Reynolds, A., Strickland, L., Gretton, M., & Matzke, D. (2019). Dynamic models of choice. Behavior Research Methods, 51, 961–985. 29959755 10.3758/s13428-018-1067-y

[ref13] Hoffman, S. N., Taylor, C. T., Campbell-Sills, L., Thomas, M. L., Sun, X., Naifeh, J. A., … Stein, M. B. (2022). Association between neurocognitive functioning and suicide attempts in U. S. army soldiers. Journal of Psychiatric Research, 145, 294–301. 33190841 10.1016/j.jpsychires.2020.11.012 PMC8102646

[ref14] Huang-Pollock, C., Ratcliff, R., McKoon, G., Roule, A., Warner, T., Feldman, J., & Wise, S. (2020). A diffusion model analysis of sustained attention in children with attention deficit hyperactivity disorder. Neuropsychology, 34(6), 641–653. 32324003 10.1037/neu0000636 PMC7957836

[ref15] Interian, A., Chesin, M., Kline, A., Miller, R., St. Hill, L., Latorre, M., … Stanley, B. (2018). Use of the Columbia-Suicide Severity Rating Scale (C-SSRS) to classify suicidal behaviors. Archives of Suicide Research, 22(2), 278–294. 28598723 10.1080/13811118.2017.1334610 PMC11822705

[ref16] Interian, A., Chesin, M. S., Stanley, B., Latorre, M., St. Hill, L. M., Miller, R. B., … Kline, A. (2021). Mindfulness-based cognitive therapy for preventing suicide in military veterans: A randomized clinical trial. Journal of Clinical Psychiatry, 82(5), 20m13791. 10.4088/JCP.20m13791 PMC11071067 34464524

[ref17] Interian, A., Myers, C. E., Chesin, M. S., Kline, A., Hill, L. S., King, A. R., … Keilp, J. G. (2020, May). Towards the objective assessment of suicidal states: Some neurocognitive deficits may be temporally related to suicide attempt. Psychiatry Research, 287, 112624. 31727438 10.1016/j.psychres.2019.112624 PMC7165019

[ref18] Karalunas, S. L., Weigard, A., & Alperin, B. (2020). Emotion-cognition interactions in attention-deficit/hyperactivity disorder: Increased early attention capture and weakened attentional control in emotional contexts. Biological Psychiatry: Cognitive Neuroscience and Neuroimaging, 5, 520–529. 32198002 10.1016/j.bpsc.2019.12.021 PMC7224233

[ref19] Katz, I. (2012). Lessons learned from mental health enhancement and suicide prevention activities in the veterans health administration. American Journal of Public Health, 102(Supplement 1), S14–S16. 22390589 10.2105/AJPH.2011.300582 PMC3496438

[ref20] Keilp, J. G., Beers, S. R., Burke, A. K., Melhem, N. M., Oquendo, M. A., Brent, D. A., & Mann, J. J. (2014a). Neuropsychological deficits in past suicide attempters with varying levels of depression severity. Psychological Medicine, 44(14), 2965–2974. 25066266 10.1017/S0033291714000786 PMC5724375

[ref21] Keilp, J. G., Sackeim, H. A., & Mann, J. J. (2005). Correlates of trait impulsiveness in performance measures and neuropsychological tests. Psychiatry Research, 135, 191–201. 15996748 10.1016/j.psychres.2005.03.006

[ref22] Keilp, J. G., Wyatt, G., Gorlyn, M., Oquendo, M. A., Burke, A. K., & Mann, J. J. (2014b). Intact alternation performance in high lethality suicide attempters. Psychiatry Research, 219(1), 129–136. 24878299 10.1016/j.psychres.2014.04.050 PMC4410782

[ref23] Kelly, S P, & Connell, R G. (2013). The neural process underlying perceptual decision making in humans: recent progress and future directions. Journal of Physiology, 109(1-3), 27–37. 10.1016/j.jphysparis.2014.08.003 25204272

[ref24] Kline, A., Chesin, M., Latorre, M., Miller, R., St. Hill, L., Shcherbakov, A., … Interian, A. (2016). Rationale and study design of a trial of mindfulness-based cognitive therapy for preventing suicidal behavior (MBCT-S) in military veterans. Contemporary Clinical Trials, 50, 245–252. 27592123 10.1016/j.cct.2016.08.015

[ref25] Le, T. P., Moscardini, E., Cowan, T., Elvevåg, B., Holmlund, T. B., Foltz, P. W., … Cohen, A. S. (2021). Predicting self-injurious thoughts in daily life using ambulatory assessment of state cognition. Journal of Psychiatric Research, 138, 335–341. 33895607 10.1016/j.jpsychires.2021.04.013

[ref26] Lerche, V., & Voss, A. (2019). Experimental validation of the diffusion model based on a slow response time paradigm. Psychological Research, 83(6), 1194–1209. 29224184 10.1007/s00426-017-0945-8

[ref27] MacLeod, C. (1991). Half a century of research on the Stroop effect: An integrative review. Psychological Bulletin, 109, 163–203. 2034749 10.1037/0033-2909.109.2.163

[ref28] Mann, J. J., Arango, V. A., Avenevoli, S., Brent, D. A., Champagne, F. A., Clayton, P., … Wenzel, A. (2009). Candidate endophenotypes for genetic studies of suicidal behavior. Biological Psychiatry, 65(7), 556–563. 19201395 10.1016/j.biopsych.2008.11.021 PMC3271953

[ref29] Millner, A. J., Robinaugh, D. J., & Nock, M. K. (2020). Advancing the understanding of suicide: The need for formal theory and rigorous descriptive research. Trends in Cognitive Sciences, 24(9), 704–716. 32680678 10.1016/j.tics.2020.06.007 PMC7429350

[ref30] Moore, T. M., Gur, R. C., Thomas, M. L., Brown, G. G., Nock, M. K., Savitt, A. P., … Stein, M. B. (2019). Development, administration, and structural validity of a brief, computerized neurocognitive battery: Results from the army study to assess risk and resilience in servicemembers. Assessment, 26(1), 125–143. 28135828 10.1177/1073191116689820 PMC6585444

[ref31] Oquendo, M. A., Sullivan, G. M., Sudol, K., Baca-Garcia, E., Stanley, B. H., Sublette, M. E., & Mann, J. J. (2014). Toward a biosignature for suicide. American Journal of Psychiatry, 171, 1259–1277. 25263730 10.1176/appi.ajp.2014.14020194 PMC4356635

[ref32] Peters, J., Vega, T., Weinstein, D., Mitchell, J., & Kayser, A. (2020). Dopamine and risky decision-making in gambling disorder. eNeuro, 7(3), ENEURO.0461-0419.2020. 10.1523/ENEURO.0461-19.2020 PMC7294471 32341121

[ref33] Polich, J. (2007). Updating P300: an integrative theory of P3a and P3b. Clinical Neurophysiology, 118(10), 2128–2148. 17573239 10.1016/j.clinph.2007.04.019 PMC2715154

[ref34] Posner, K., Brodsky, B., Yershova, K. V., Buchanan, J., & Mann, J. (2014). The classification of suicide behavior. In M. K. Nock (Ed.), The Oxford handbook of suicide and self-injury (pp. 7–22). New York: Oxford University Press.

[ref35] Posner, K., Brown, G., Stanley, B., Brent, D. A., Yershova, K. V., Oquendo, M. A., … Mann, J. J. (2011). The Columbia-Suicide Severity Rating Scale (C-SSRS): Initial validity and internal consistency findings from three multi-site studies with adolescents and adults. American Journal of Psychiatry, 168(12), 1266–1277. 22193671 10.1176/appi.ajp.2011.10111704 PMC3893686

[ref36] Ratcliff, R. (1993). Methods for dealing with reaction time outliers. Psychological Bulletin, 114(3), 510–532. 8272468 10.1037/0033-2909.114.3.510

[ref37] Ratcliff, R., Huang-Pollock, C., & McKoon, G. (2018). Modeling individual differences in the Go/No-Go task with a diffusion model. Decision, 5(1), 42–62. 29404378 10.1037/dec0000065 PMC5796558

[ref38] R Core Team. (2017). R: A language and environment for statistical computing. R Foundation for Statistical Computing. Vienna, Austria. https://www.R-project.org/.

[ref39] Richard-Devantoy, S., Berlim, M. T., & Jollant, F. (2014). A meta-analysis of neuropsychological markers of vulnerability to suicidal behavior in mood disorders. Psychological Medicine, 44(8), 1663–1673. 24016405 10.1017/S0033291713002304

[ref40] Richard-Devantoy, S., Jollant, F., Kefi, Z., Turecki, G., Olié, J. P., Annweiler, C., … Le Gall, D. (2012). Deficit of cognitive inhibition in depressed elderly: A neurocognitive marker of suicidal risk. Journal of Affective Disorders, 140(2), 193–199. 22464009 10.1016/j.jad.2012.03.006

[ref41] Stanley, B., & Brown, G. K. (2012). Safety planning intervention: A brief intervention to mitigate suicide risk. Cognitive and Behavioral Practice, 19(2), 256–264.

[ref42] Stanley, B., Brown, G. K., Brenner, L. A., Galfalvy, H. C., Currier, G. W., Knox, K. L., … Green, K. L. (2018). Comparison of the safety planning intervention with follow-up vs usual care of suicidal patients treated in the emergency department. JAMA Psychiatry, 75(9), 894–900. 29998307 10.1001/jamapsychiatry.2018.1776 PMC6142908

[ref43] Stroop, J. R. (1935). Studies of interference in serial verbal reactions. Journal of Experimental Psychology, 18(6), 643–662.

[ref44] van Heeringen, K., & Mann, J. J. (2014). The neurobiology of suicide. The Lancet Psychiatry, 1, 63–72. 26360403 10.1016/S2215-0366(14)70220-2

[ref45] Weigard, A., Heathcote, A., & Sripada, C. (2019). Modeling the effects of methylphenidate on interference and evidence accumulation processes using the conflict linear ballistic accumulator. Psychopharmacology, 236(8), 2501–2512. 31302719 10.1007/s00213-019-05316-x PMC6697566

[ref46] Weigard, A., & Huang-Pollock, C. (2017). The role of speed in ADHD-related working memory deficits: A time-based resource-sharing and diffusion model account. Clinical Psychological Science, 5(2), 195–211. 28533945 10.1177/2167702616668320 PMC5437983

[ref47] Weigard, A., & Sripada, C. (2021). Task-general efficiency of evidence accumulation as a computationally defined neurocognitive trait: Implications for clinical neuroscience. Biological Psychiatry: Global Open Science, 1(1), 5–15. 35317408 10.1016/j.bpsgos.2021.02.001 PMC8936715

[ref48] Westheide, J., Quednow, B. B., Kuhn, K.-U., Hoppe, C., Cooper-Mahkorn, D., Hawellek, B., … Wagner, M. (2008). Executive performance of depressed suicide attempters: The role of suicidal ideation. European Archives of Psychiatry and Clinical Neuroscience, 258, 414–421. 18330667 10.1007/s00406-008-0811-1

[ref49] Wright, L., Lipszyc, J., Dupuis, A., Thayapararajah, S., & Schachar, R. (2014). Response inhibition and psychopathology: A meta-analysis of go/no-go task performance. Journal of Abnormal Psychology, 123(2), 429–439. 24731074 10.1037/a0036295

[ref50] Zhang, J., Rittman, T., Nombela, C., Fois, A., Coyle-Gilchrist, I., Barker, R. A., … Rowe, J. B. (2016). Different decision deficits impair response inhibition in progressive supranuclear palsy and Parkinson's disease. Brain, 139(Pt 1), 161–173. 26582559 10.1093/brain/awv331 PMC4949391

