# Supplementary material for: Improving the prospective prediction of a near-term suicide attempt in veterans at risk for suicide, using a go/no-go task
Source: Psychol Med. 2022 Jul 28;53(9):4245–54. doi: 10.1017/S0033291722001003 (PMC9883589; doi:10.1017/S0033291722001003)
Supplement: Supplementary file 1 [file S0033291722001003sup.zip › S0033291722001003sup001.docx]

# Improving the Prospective Prediction of a Near-Term Suicide Attempt in Veterans at Risk for Suicide, Using a Go/No-Go Task

# Supplemental Table: Detailed Behavioral and LBA Results

Supplemental table shows detailed behavioral and modeling results, for total sample of 284 datafiles, and broken down by outcome group, based on the 90 days following the Go/No-go (GNG) data collection.

|  | NoSE Group | | | OtherSE Group | | | ASA Group | | | Total Sample | | |
| --- | --- | --- | --- | --- | --- | --- | --- | --- | --- | --- | --- | --- |
|  | N | Mean | SD | N | Mean | SD | N | Mean | SD | N | Mean | SD |
| **Go/No-go (GNG) Task** |  |  |  |  |  |  |  |  |  |  |  |  |
| Total trials completed | 237 | 224.20 | 9.35 | 29 | 225.00 | 0.00 | 18 | 225.00 | 0.00 | 284 | 224.33 | 8.55 |
| # short-RT trials dropped | 237 | 0.76 | 3.73 | 29 | 2.90 | 7.98 | 18 | 0.50 | 0.99 | 284 | 0.96 | 4.29 |
| # long-RT trials dropped | 237 | 0.78 | 1.72 | 29 | 0.76 | 1.06 | 18 | 1.17 | 2.96 | 284 | 0.80 | 1.76 |
| # trials analyzed | 237 | 222.66 | 10.21 | 29 | 221.34 | 8.68 | 18 | 223.33 | 3.41 | 284 | 222.57 | 9.76 |
| % misses | 237 | 5.14 | 10.84 | 29 | 4.42 | 5.70 | 18 | 18.99 | 27.36 | 284 | 5.95 | 12.56 |
| % false alarms (FA) | 237 | 12.41 | 10.45 | 29 | 18.44 | 15.85 | 18 | 11.19 | 8.16 | 284 | 12.94 | 11.11 |
| % FA, identity foils | 237 | 20.93 | 16.63 | 29 | 28.09 | 19.81 | 18 | 15.91 | 11.22 | 284 | 21.34 | 16.85 |
| % FA, location foils | 237 | 5.57 | 7.90 | 29 | 10.73 | 14.47 | 18 | 7.42 | 8.60 | 284 | 6.21 | 8.93 |
| Mean RT (sec): hits | 237 | 0.44 | 0.07 | 29 | 0.42 | 0.06 | 18 | 0.45 | 0.07 | 284 | 0.43 | 0.07 |
| Mean RT (sec): all FA | 229 | 0.38 | 0.08 | 28 | 0.36 | 0.06 | 18 | 0.40 | 0.08 | 275 | 0.38 | 0.08 |
| Mean RT: FA to identity foils | 221 | 0.36 | 0.06 | 28 | 0.35 | 0.06 | 17 | 0.35 | 0.04 | 266 | 0.36 | 0.06 |
| Mean RT: FA to location foils | 165 | 0.43 | 0.11 | 21 | 0.39 | 0.09 | 14 | 0.48 | 0.17 | 200 | 0.43 | 0.11 |
| RT variance: hits | 237 | 0.01 | 0.01 | 29 | 0.01 | 0.01 | 18 | 0.01 | 0.01 | 284 | 0.01 | 0.01 |
| RT variance: FA | 217 | 0.01 | 0.02 | 28 | 0.01 | 0.01 | 17 | 0.02 | 0.02 | 262 | 0.01 | 0.02 |
| RT variance: identity foils | 207 | 0.01 | 0.02 | 27 | 0.00 | 0.01 | 15 | 0.01 | 0.01 | 249 | 0.01 | 0.02 |
| RT variance: location foils | 122 | 0.02 | 0.02 | 16 | 0.01 | 0.02 | 10 | 0.02 | 0.02 | 148 | 0.02 | 0.02 |
| **Linear Ballistic Accumulator (LBA) Model** |  |  |  |  |  |  |  |  |  |  |  |  |
| *A* | 217 | 0.71 | 0.37 | 28 | 0.62 | 0.39 | 17 | 0.81 | 0.48 | 262 | 0.71 | 0.38 |
| *B_No-go_* | 217 | 0.8 | 0.27 | 28 | 0.82 | 0.25 | 17 | 0.81 | 0.34 | 262 | 0.81 | 0.27 |
| *B_Go_* | 217 | 0.75 | 0.3 | 28 | 0.63 | 0.3 | 17 | 0.66 | 0.25 | 262 | 0.73 | 0.30 |
| *v_foil-No-go_* (correct withhold) | 217 | 5.4 | 1.34 | 28 | 5.27 | 1.35 | 17 | 5.23 | 1.63 | 262 | 5.37 | 1.36 |
| *v_target-Go_* (hit) | 217 | 3.86 | 0.83 | 28 | 3.59 | 1.05 | 17 | 3.43 | 1.03 | 262 | 3.80 | 0.88 |
| *v_foil-Go_* (false alarm) | 217 | 2.89 | 1.07 | 28 | 2.63 | 0.99 | 17 | 2.29 | 1.09 | 262 | 2.82 | 1.07 |
| *v_target-No-go_* (miss) | 217 | 0.74 | 0.55 | 28 | 0.84 | 0.56 | 17 | 1.09 | 1.11 | 262 | 0.77 | 0.60 |
| *t0* | 217 | 0.14 | 0.03 | 28 | 0.14 | 0.03 | 17 | 0.14 | 0.03 | 262 | 0.14 | 0.03 |
| Response bias for Go | 217 | 5.38 | 26.90 | 28 | 19.50 | 23.75 | 17 | 14.53 | 26.73 | 262 | 7.48 | 26.89 |
| Decisional efficiency: foils | 217 | 2.5 | 1.17 | 28 | 2.64 | 1.13 | 17 | 2.93 | 1.32 | 262 | 2.55 | 1.18 |
| Decisional efficiency: targets | 217 | 3.12 | 1.14 | 28 | 2.74 | 1.29 | 17 | 2.33 | 2.04 | 262 | 3.03 | 1.24 |
| Gelman-Rubin R̂ | 217 | 1.08 | 0.01 | 28 | 1.08 | 0.01 | 17 | 1.08 | 0.02 | 262 | 1.08 | 0.01 |
| # Iterations to convergence | 217 | 748.03 | 153.72 | 28 | 771.14 | 178.92 | 17 | 751.65 | 144.63 | 262 | 750.73 | 155.57 |
| Minimum Effective Size | 217 | 649.64 | 96.62 | 28 | 639.07 | 100.42 | 17 | 642.35 | 106.51 | 262 | 648.04 | 97.36 |
| Mean deviance | 217 | -205.2 | 132.92 | 28 | -215.94 | 145.25 | 17 | -128.08 | 191.24 | 262 | -201.35 | 139.39 |
| WAIC | 217 | -199.25 | 132.96 | 28 | -210.36 | 145.2 | 17 | -122.16 | 191.12 | 262 | -195.44 | 139.42 |
| **Stroop Task** |  |  |  |  |  |  |  |  |  |  |  |  |
| Total trials completed | 236 | 107.27 | 6.66 | 29 | 108 | 0 | 17 | 108 | 0 | 282 | 107.39 | 6.09 |
| # short-RT trials dropped | 236 | 0.16 | 1.38 | 29 | 0.03 | 0.19 | 17 | 0 | 0 | 282 | 0.13 | 1.27 |
| # long-RT trials dropped | 236 | 0.15 | 0.73 | 29 | 0.03 | 0.19 | 17 | 0.53 | 1.37 | 282 | 0.16 | 0.75 |
| # trials analyzed | 236 | 106.96 | 6.97 | 29 | 107.93 | 0.37 | 17 | 107.47 | 1.37 | 282 | 107.09 | 6.39 |
| d-score | 236 | 0.19 | 0.14 | 29 | 0.19 | 0.17 | 17 | 0.24 | 0.18 | 282 | 0.19 | 0.15 |
| Mean RT (msec), Congruent | 236 | 1169.05 | 367.2 | 29 | 1132.75 | 315.91 | 17 | 1471.86 | 673.13 | 282 | 1183.58 | 392.40 |
| Mean RT (msec), Incongruent | 236 | 1390.89 | 462.94 | 29 | 1347.46 | 411.16 | 17 | 1858.46 | 1004.84 | 282 | 1414.61 | 516.16 |

*Notes: ASA=actual suicide attempt, OtherSE=other suicide-related event (SE) excluding ASA, noSE=no SE; RT=reaction time; FA=false alarms. Linear Ballistic Accumulator (LBA) model parameters A=start point variability; B_No-go_/B_Go_ = boundary offset for No-go and Go accumulators; v=average slope for each combination of stimulus type (target, foil) and response (Go, No-go); all reported as means (averages) of medians of the posterior distribution. Response bias for Go = 100*(B_No-go_ - B_Go_); decisional efficiency for foils = v_foil-No-go_ - v_foil-Go_; decisional efficiency for targets = v_target-Go_ - v_target-No-go_; WAIC=Wantanabe-Akaike Information Criterion; Stroop task d-score = (mean RT for Incongruent – mean RT for Congruent)/(mean RT for Congruent). Contemporaneous Stroop data were not available at all GNG testing sessions. RT mean for false alarms could only be calculated for GNG datafiles with 1+ false alarms; RT variance could only be calculated for, and LBA model could only be applied to, GNG datafiles containing 2+ false alarms. Note also that each unique participant could have contributed more than one datafile to this analysis; within-subject effects are not controlled for in this table, but were included in the GEE models.*
