# Supplementary material for: Improving the prospective prediction of a near-term suicide attempt in veterans at risk for suicide, using a go/no-go task
Source: Psychol Med. 2022 Jul 28;53(9):4245–54. doi: 10.1017/S0033291722001003 (PMC9883589; doi:10.1017/S0033291722001003)
Supplement: Supplementary file 1 [file S0033291722001003sup.zip › S0033291722001003sup002.docx]

# Improving the Prospective Prediction of a Near-Term Suicide Attempt in Veterans at Risk for Suicide, Using a Go/No-Go Task:

# Supplemental Text

## Contents:

- RCT Participant Details
- LBA Modeling Methods
- References

# RCT Participant Details

Full details of the randomized clinical trial (RCT) are provided in (Interian et al., 2021). In brief, participants were recruited from December 2013 through March 2018 from two Veterans Health Administration (VHA) Medical Centers. All new psychiatric inpatient admissions were screened via electronic medical records, as well as monitoring of outpatients designated as high-risk for suicide.

Inclusion criteria for the RCT were:

1. Age at least 18 years
2. Significant suicide risk in prior 30 days, e.g., suicide behavior (SB) or suicidal ideation (SI) with intent to attempt suicide.
3. An actual, aborted or interrupted suicide attempt in the prior 12 months, or placement on the VHA high-risk for suicide list (HRSL). Placement on VHA HRSL is determined by clinical judgment on the part of the VHA Suicide Prevention Coordinator(s), based on evaluation of risk factors such as history of past suicide attempts, recent discharge from an inpatient mental health using, and the presence or absence of warning signs such as threatening to hurt oneself, seeking suicide methods, and talking or writing about death or dying.

Suicide behaviors included actual suicide attempt (ASA) defined as deliberate self-directed violence with injury or potential injury and with explicit or implicit suicidal intent (Matarazzo et al., 2013); interrupted or aborted suicide attempt; or preparatory behavior (e.g. acquiring means such as pills or firearm, writing a note). These were assessed using the clinician-administered Columbia Suicide Severity Rating Scale (C-SSRS; Posner et al., 2011); interrater agreement was 0.89 for suicide behavior classifications, using a blind panel consensus process to classify ambiguous cases (Interian et al., 2018). Suicidal ideation was measured using the clinician-administered Scale for Suicide Ideation (SSI; Beck et al., 1979).

Exclusion criteria for the RCT were:

1. Cognitive impairment likely to limit therapeutic benefit from cognitive therapy
2. Severe psychotic symptoms, assessed using the Mini International Neuropsychiatric Interview (MINI, v. 5.0.0) (Sheehan et al., 1998).
3. Disorganized or disruptive behavior
4. Medical instability
5. 2 or more sessions of psychotherapy with a mindfulness-based intervention in prior year.

History of traumatic brain injury (TBI) was assessed via self-report with a single item inquiring about lifetime history of head injury involving lack of consciousness, amnesia, seizures, or headaches, and was coded as a binary variable (yes/no) but was not an inclusion/exclusion criterion.

RCT participants were randomized to a treatment group or control group. The control group received enhanced treatment-as-usual (eTAU) for suicide, VHA’s enhanced program for Veterans at high-risk for suicide (Katz, 2012). In brief, VHA Suicide Prevention Coordinators provided study participants the Safety Planning Intervention (SPI) (Stanley & Brown, 2012), monitored their clinical status, and made attempts to support mental health treatment engagement. Participants in the treatment group received eTAU plus Mindfulness-Based Cognitive Therapy adapted for Suicide (MBCT-S). MBCT-S began with two individual sessions focused on elaborating the SPI safety plan (Stanley & Brown, 2012), followed by 8 group-based mindfulness sessions, followed by optional monthly booster sessions.

For participants in the RCT, assessments including neurocognitive testing occurred at enrollment (baseline, prior to treatment), 3 months later (post-treatment), and again at 6 months and 12 months post baseline. There was also a clinical assessment mid-treatment (approx. 6 weeks post baseline) but neurocognitive tests were not administered at this timepoint.

The RCT primary outcome was suicide events (SE), which included actual suicide attempts (ASA), aborted or interrupted suicide attempts, suicide preparatory behaviors, or suicidal ideation resulting in acute psychiatric hospitalization or emergency department (ED) visit. Suicide behaviors including ASA, interrupted/aborted attempts, and preparatory behavior were based on C-SSRS from all available timepoints. Medical record review was used to determine suicide-related hospitalizations not associated with suicidal behavior; in one case, chart review identified an ASA that occurred within the 90-day follow-up window but could not be confirmed by subsequent C-SSRS due to patient lost to follow-up.

# LBA Modeling Methods

The linear ballistic accumulator model (LBA) (Brown & Heathcote, 2008; Donkin et al., 2011), is a mathematically simpler (more computationally tractable) alternative to the drift diffusion model (DDM) (Ratcliff, 1978; Ratcliff & McKoon, 2008; Ratcliff et al., 2016). The LBA assumes separate evidence accumulation processes (“racers”) for each response alternative, where the first racer to reach an evidence threshold “wins” and triggers the corresponding response (see Figure 2A). There is variability across trials in starting point and in slope of evidence accumulation, but once started, each racer’s trajectory is linear and fixed (hence “ballistic”). This simplification allows complete analytic solutions for choices between any number of response alternatives. Brown and Heathcote (2008) showed that the LBA model could account for empirical phenomena including RT distribution shape, speed-accuracy tradeoffs, and the relative speed of correct vs. incorrect responses depending on task instructions. Parameters estimated by the DDM and LBA are often highly correlated (e.g., Brown & Heathcote, 2008; Donkin et al., 2011); however, the LBA may be preferred in situations such as the GNG where No-go responses are unobservable and RT must be imputed. The LBA has been applied to GNG data to capture different cognitive processes underlying GNG performance in disorders such as externalizing psychopathology (Endres et al., 2014) and attention-deficit/hyperactivity disorder (Karalunas et al., 2020).

Here, we implemented a Bayesian version of the LBA, using the Dynamic Models of Choice (DMC) package v. 190819 (Heathcote et al., 2019) and base R functions (R Core Team, 2017). DMC provides the lba_Bgng.R implementation of LBA for the Go/No-go task, which assumes separate accumulators for No-go and Go responses (as schematized in Figure 2A), and calculates likelihood of Go RTs given the defective probability density function for Go responses predicted by the model (Turner et al., 2013); for No-go RTs, the likelihood is calculated by integrating the defective probability density function for No-go responses and assigning the same likelihood value to all No-go responses in a given condition (Karalunas et al., 2020). Model-building methods generally followed Karalunas et al. (2020), except that we did not include case-outcome information in the model-building procedure, since the ultimate goal here was to predict those case outcomes, and including that information *a priori* would therefore have been circular logic.

## LBA Modeling Methods and Posterior Estimates

For each GNG datafile containing at least 2 false alarms (and at least 2 hits), so that RT variance can be calculated for each stimulus type (n=262 of the 284 datafiles in our sample), Markov Chain Monte Carlo (MCMC) simulations were used to sample from posterior distributions of 8 free parameters (refer Figure 2A): non-decision time (*t0*), starting point variability (*A*), boundary offset (*B_No-go_* and *B_Go_* for the No-go and Go accumulators, respectively), and mean slope parameters *v* for each combination of stimulus type (foil vs. target) and accumulator (No-go vs. Go). Following (Karalunas et al., 2020), for simplicity, slope variability (*sv*) was held constant at 1 and *t0* variability (*st0*) was held constant at 0.

In defining priors for the 8 free parameters, we followed Karalunas et al. (2020) in assuming that *A*, *B_No-go_*, *B_Go_*, and mean slopes *v* were drawn from normal distributions (*A* and *B* values truncated at 0 to avoid negative values); values for *t0* were drawn from a uniform distribution in the range U[0.1, 1.0]. Table S1 shows mean/SD of the priors for each parameter.

| **Parameter** | **Interpretation** | **Prior mean (SD)** |
| --- | --- | --- |
| *A* | Starting point variability | 0.3 (1) |
| *B_No-go_* | Response offset, No-go accumulator | 0.5 (1) |
| *B_Go_* | Response offset, Go accumulator | 0.5 (1) |
| *v_target-Go_* | Mean slope for evidence accumulation to target in Go accumulator  (resulting in correct hit to target) | 1 (3) |
| *v_foil-No-go_* | Mean slope for evidence accumulation to foil in No-go accumulator  (resulting in correct withholding of response) | 1 (3) |
| *v_target-No-go_* | Mean slope for evidence accumulation to target in No-go accumulator (resulting in incorrect missed response to target) | -1 (3) |
| *v_foil-Go_* | Mean slope for evidence accumulation to foil in Go accumulator  (resulting in incorrect false alarm to foil) | -1 (3) |
| *t0* (in sec) | Non-decision time (stimulus encoding + motor response execution) | 0.55 (0.26) |

*Table S1. Priors used in the LBA model, for the 8 free parameters (A*, *B_No-go_*, *B_Go_* *drawn* *from truncated normal distributions, mean slopes v from normal distributions, t0 from uniform distribution). See also Figure 2A.*

Chains were constructed and iteratively checked, using DMC’s RUN-dmc() routine (Heathcote et al., 2019) until convergence and stability criteria were met. In brief, for each datafile, starting values were generated for 24 chains (i.e., 3x number of free parameters), and an initial set of 500 samples was generated. Any “stuck” chains, defined based on deviation >10 of mean chain log-likelihood from median of means of other chains, were discarded and re-run; stationarity was checked by comparing the first third and last third of each chain, throwing away the first third of samples if checks fail and adding the same number of samples; mixing was assessed by the Gelman-Rubin R̂ statistic (Brooks & Gelman, 1998; Gelman & Rubin, 1992), using criterion of R̂<1.1. Checks were made repeatedly until stationarity and convergence criteria were reached and a minimum number of 2000 samples were obtained (or a maximum of 10 attempts was exhausted). For successful runs, the wrapper recorded number of iterations, multivariate Gelman-Rubin R̂, effective sample size (taking into account autocorrelation between samples), and the posteriors obtained (mean/SD and median/95% CI) for each parameter. Convergence was assessed by (1) visual inspection of chains (see Figure S1A obtained from one example datafile); (2) Gelman-Rubin R̂<1.1 for all parameters; (3) no “stuck chains” identified by DMC’s pick.stuck.chains() function; and (4) effective size>500 for all parameters. Representative results for one GNG datafile (668 samples per chain, all R̂≤1.06, all effective size>=585) are shown in Figure S1B.

Posterior predictive samples were generated from the posterior estimates; posterior predictive plots, displaying degraded probability density function and joint cumulative distribution function for the empirical and predicted RT data at 5 quantiles (.1, .3, .5, .7, .9), were generated to visually assess model fit (results from a representative GNG datafile appear in Figure S1C-F).

Each datafile took ~1-2 min to run (depending on iterations required before convergence) using R v. 3.6.2 on a Dell desktop PC (Intel Core 3.20 GHz with 16 GB RAM) under Windows 10 Enterprise (single core). Convergence and stationarity criteria were met for all datafiles except one (a noSE case); this file was re-run using RUN.dmc() and converged on the second attempt.

| A  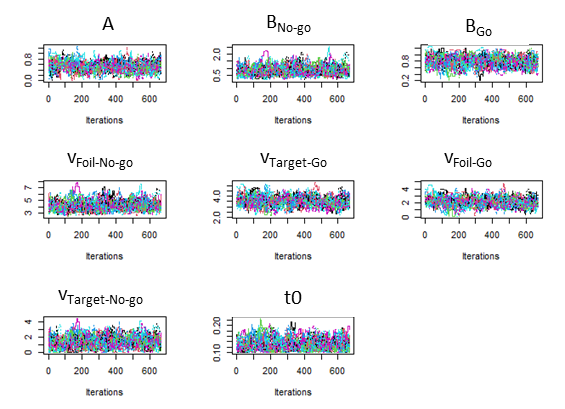 | B  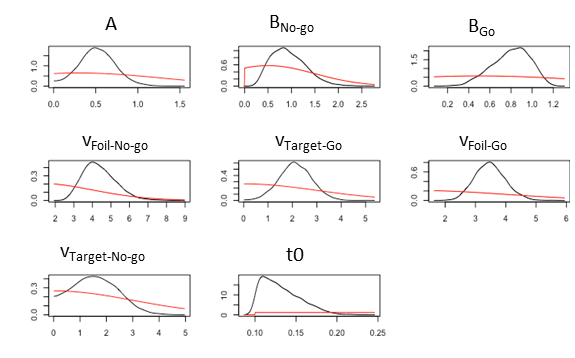 |
| --- | --- |
| C  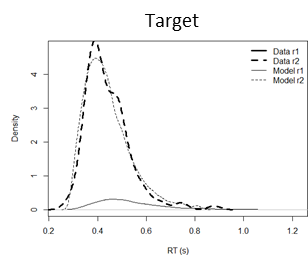 | D  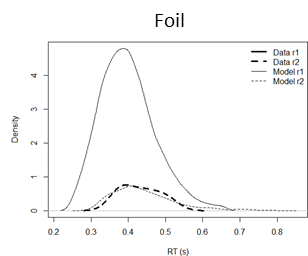 |
| E  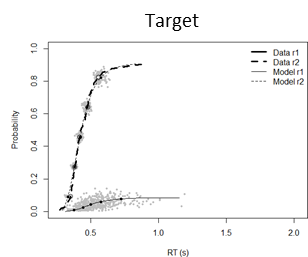 | F  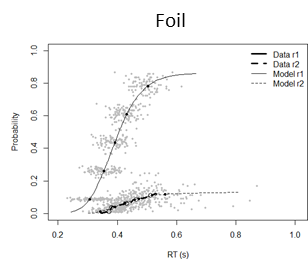 |

*Figure S1. Example results from LBA fitted to one representative GNG datafile. (A) Markov Chain Monte Carlo (MCMC) chains after discarding burn-in show characteristic appearance of “fat, flat hairy caterpillars” for each parameter, indicating good mixing and stability. (B) Posterior distributions for each LBA parameter (black lines) are unimodal and more tightly specified compared to vaguely-defined priors (red lines). (C,D) Degraded probability density functions of reaction time (RT) distributions to targets and foils as predicted by the LBA based on posterior parameter estimates (thin solid lines=No-go responses; thin dashed lines=Go responses) and original data (heavy dashed lines=Go responses; note that RTs for No-go responses are unobserved in the data). (E,F) Degraded cumulative distribution function of RTs to targets and foils as predicted by LBA vs. those observed in the data. Plots drawn using DMC’s plot.dmc() and plot.pp.dmc() functions.*

The resulting posteriors (median of posterior distribution) were recorded for each GNG datafile; the Supplemental Table reports posterior estimates (medians) as well as iterations to convergence, Gelman-Rubin R̂, and minimum effective size, for the complete set of 262 GNG datafiles, and also broken down each 90-day outcome group.

Figure S2 shows correlations between posterior estimates for each parameter across the 262 datafiles; as expected with the LBA, some parameter estimates are correlated (e.g. response offset in each accumulator and mean slope for the corresponding correct response: B_No-go_ with v_foil-No-go_ and B_Go_ with v_target-Go_), such correlation does not necessarily mean that the estimates are unreliable (Heathcote et al., 2019).

| 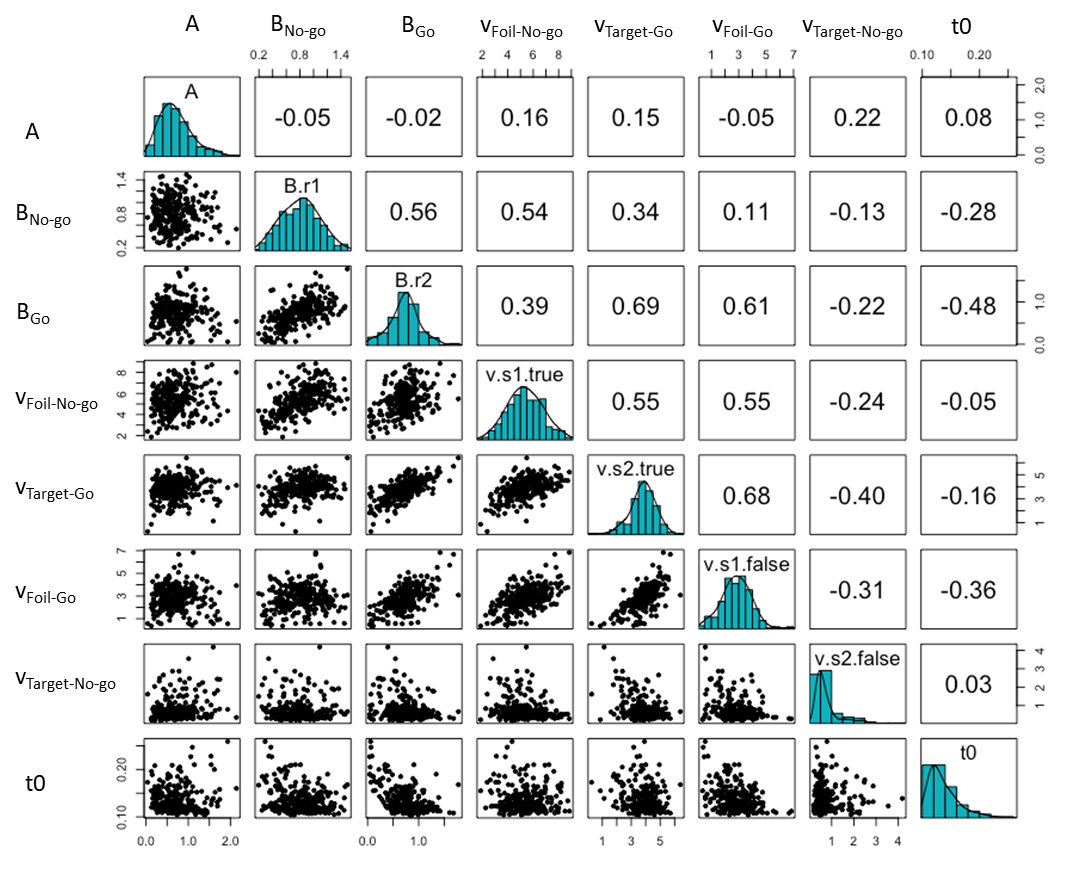 | *Figure S2. Scatterplots illustrating correlations among estimated parameters, using median of the posterior distribution as a point estimate for each GNG datafile. Upper panels show Pearson’s r for each pair of parameter estimates; diagonal shows density plot of medians for each parameter.* |
| --- | --- |

In general, posterior estimates appeared reasonable. For example, the boundary offset for the No-go accumulator tended to be larger than that for the Go accumulator, indicating a response bias favoring the (more frequently correct) Go response. Posterior estimates for the mean slopes *v* associated with correct responses (v_foil-No-go_ and v_target-Go_) were larger than those associated with incorrect responses (v_foil-Go_ and v_target-No-go_), indicating faster evidence accumulation for correct than incorrect responses. Finally, the posteriors for non-decision time (*t0*) averaged about 140 msec, which would represent about one-third of the total RT (which averaged about 430 msec for hits and 380 msec for false alarms). In general, then, the posteriors appeared consistent with what is known and expected about the GNG task.

## Replicability Tests

To assess replicability of the results from the 8-parameter LBA, we also re-ran the complete set of GNG files, using the same 8-parameter LBA model, but with different random start values; Figure S3A shows extremely high correlation between the median estimates for each parameter value obtained across the two runs (Supplemental Figure S3A; all r>0.99 except *t0* r>0.97). We also re-ran the LBA on the complete set of GNG files without any data cleaning (i.e., without excluding very short- and very-long RT trials); Figure S3B shows reasonably high correlation between parameter estimates obtained with and without data cleansing (all 0.85<r<0.98), indicating the results were relatively robust to decisions about whether to exclude outlier trials.

| (A)  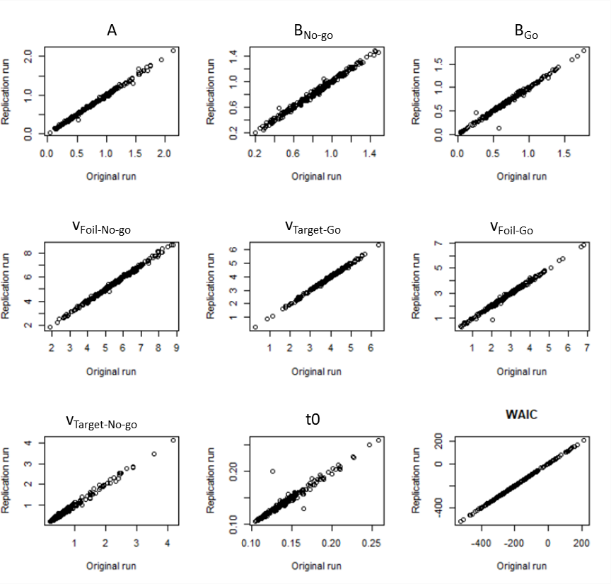 | (B)  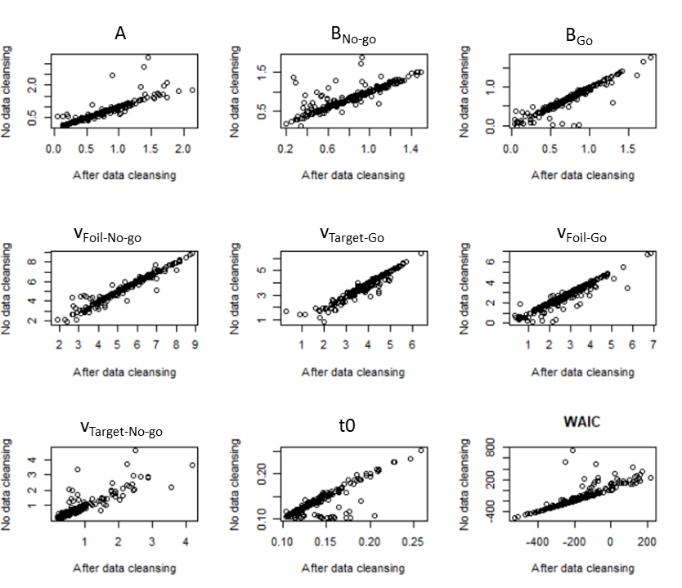 |
| --- | --- |

*Figure S3. Replicability of LBA estimates. (A) The LBA model was run a second time on all 262 GNG datasets, using the same LBA model but different random starting points, to assess replicability. There was extremely high consistency in parameter estimates obtained in the original and replication samples (all r>0.99 except t0, r>0.97), as well as in model-fit metrics such as Watanabe-Akaike Information Criterion (WAIC). (B) To assess sensitivity of LBA estimates to data cleansing, we also re-ran the LBA on all 262 datasets without data cleansing (i.e., without excluding very short- and very-long RT trials). There was reasonably high correlation between parameter estimates obtained with and without data cleansing (all 0.85<r<0.98).*

## Simulation Study and Parameter Recovery Tests

As a further check on model validity, we conducted a parameter recovery test, following previously-used methods (Karalunas et al., 2020; Lerche et al., 2017; Ratcliff & Childers, 2015; White et al., 2018), to assess whether parameter values could be accurately recovered. We generated 262 simulated datasets, each including 81 foil and 144 target trials as in the behavioral data; this included 17 simulated datasets using the ASA group medians as priors, 28 simulated datasets using the OtherSE group medians as priors and 262 using the noSE group medians as priors. As in the behavioral data, RT was undefined for trials with No-go responses.

Figure S4 shows that the simulated data accurately recovered key features of the behavioral data, including higher miss rate in the ASA group and higher false alarm rate in the OtherSE group.

We then used the LBA to estimate parameters for each of the 262 simulated GNG files (parameter recovery test), using the same priors and procedures as had been used with the behavioral datafiles. Posterior parameter estimates from this parameter recovery test are shown in Table S2; recovered values for the NoSE and OtherSE group were all fairly close to generating values; those from the ASA group are somewhat less accurate, as would be expected given the smaller number of datapoints (N=17 datafiles).

For all parameters, the 95% CI included the true (generating) parameter values, and the recovered parameter correctly captured group differences of higher response bias for Go responding in the OtherSE group (Figure S4C) and of lower decisional efficiency for targets in the ASA group (Figure S4E). Therefore, we concluded that the LBA model can reliably recover parameter values, which suggests that analysis of group differences in estimated parameters is interpretable.

| A  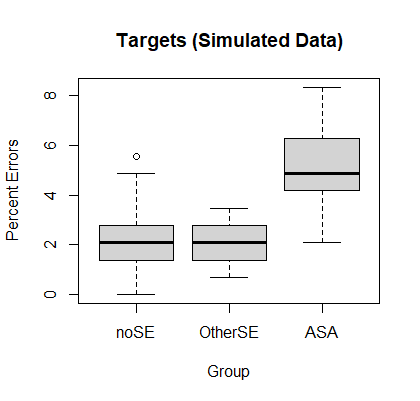 | B  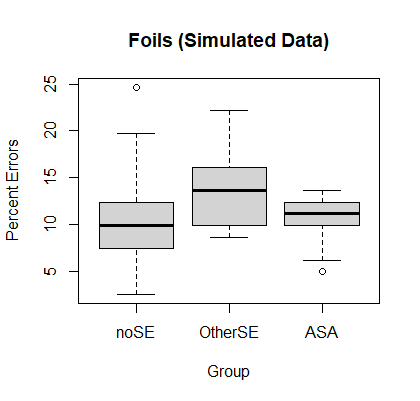 |
| --- | --- |
| C  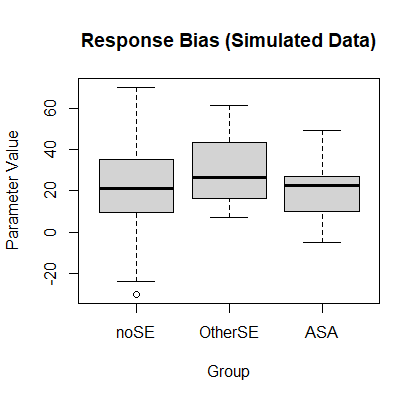 | D  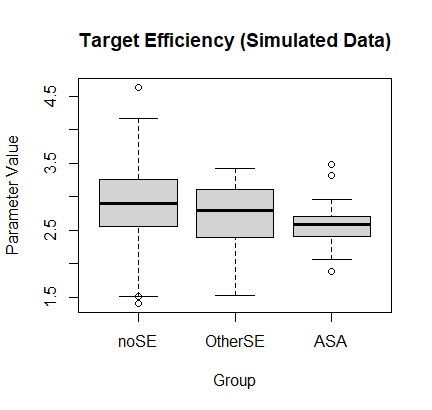 |
| E  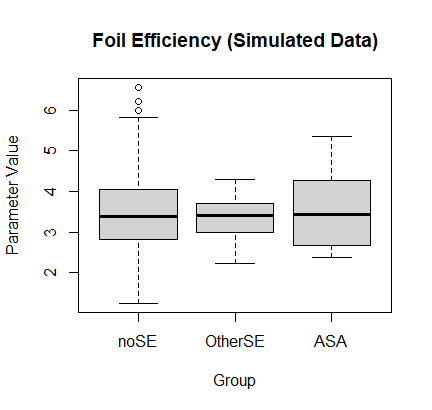 | *Figure S4. Results from simulation study and parameter recovery tests. (A,B) Accuracy rates in 262 “simulated” datafiles generated from median parameter estimates in the noSE, OtherSE, and ASA groups (group means shown in Table 1). (C,D,E) Latent parameters obtained from LBA modeling of the simulated datafiles (parameter recovery test) captured key features of the estimates obtained from the behavioral data, including increased response bias for Go responses in the OtherSE group and decreased decisional efficiency for targets in the ASA group.* |

|  | No SE Group (N=217) | | OtherSE Group (N=28) | | ASA Group (N=17) | |
| --- | --- | --- | --- | --- | --- | --- |
|  | Generating | Recovered | Generating | Recovered | Generating | Recovered |
| *A* | 0.71 | 0.70 | 0.62 | 0.65 | 0.81 | 0.87 |
| *B_No-go_* | 0.8 | 0.83 | 0.82 | 0.82 | 0.81 | 0.66 |
| *B_Go_* | 0.75 | 0.65 | 0.63 | 0.57 | 0.66 | 0.40 |
| *v_target-Go_* (hit) | 3.86 | 3.70 | 3.59 | 3.65 | 3.43 | 3.45 |
| *v_foil-No-go_* (correct withhold) | 5.4 | 5.7 | 5.27 | 5.64 | 5.23 | 5.45 |
| *v_target-No-go_* (miss) | 0.74 | 0.76 | 0.84 | 0.74 | 1.09 | 0.82 |
| *v_foil-Go_* (false alarm) | 2.89 | 2.37 | 2.63 | 2.28 | 2.29 | 1.86 |
| *t0* | 0.14 | 0.16 | 0.14 | 0.16 | 0.14 | 0.18 |

*Table S2. Medians of posterior parameter estimates recovered from the simulated datasets, averaged across the datasets in each outcome group. For comparison, generating parameters (medians of posterior parameter estimates for each outcome group in the behavioral data) are shown.*

# References

Beck, A. T., Kovacs, M., & Weissman, A. (1979). Assessment of suicidal intention: the Scale for Suicide Ideation. *Journal of Consulting and Clinical Psychology, 47*(2), 343-352.

Brooks, S. P., & Gelman, A. (1998). General methods for monitoring convergence of iterative simulations. *Journal of Computational and Graphical Statistics, 7*, 434-455.

Brown, S. D., & Heathcote, A. (2008). The simplest complete model of choice response time. *Cognitive Psychology, 57*(3), 153-178.

Donkin, C., Brown, S., Heathcote, A., & Wagenmakers, E.-J. (2011). Diffusion versus linear ballistic accumulation: Different models but the same conclusions about psychological processes? *Psychonomic Bulletin and Review, 18*, 61-69.

Endres, M. J., Donkin, C., & Finn, P. R. (2014). An information processing/associative learning account of behavioral disinhibition in externalizing psychopathology. *Experimental and Clinical Psychopharmacology, 22*(2), 122-132.

Gelman, A., & Rubin, D. B. (1992). Inference from iterative simulation using multiple sequences (with discussion). *Statistical Science, 7*, 457-511.

Heathcote, A., Lin, Y.-S., Reynolds, A., Strickland, L., Gretton, M., & Matzke, D. (2019). Dynamic models of choice. *Behavior Research Methods, 51*, 961-985.

Interian, A., Chesin, M., Kline, A., Miller, R., St. Hill, L., Latorre, M., . . . Stanley, B. (2018). Use of the Columbia-Suicide Severity Rating Scale (C-SSRS) to classify suicidal behaviors. *Archives of Suicide Research, 22*(2), 278-294.

Interian, A., Chesin, M. S., Stanley, B., Latorre, M., St. Hill, L. M., Miller, R. B., . . . Kline, A. (2021). Mindfulness-based cognitive therapy for preventing suicide in military veterans: A randomized clinical trial. *Journal of Clinical Psychiatry, 82*(5), 20m13791.

Karalunas, S. L., Weigard, A., & Alperin, B. (2020). Emotion-cognition interactions in attention-deficit/hyperactivity disorder: Increased early attention capture and weakened attentional control in emotional contexts. *Biological Psychiatry: Cognitive Neuroscience and Neuroimaging, 5*, 520-529.

Katz, I. (2012). Lessons learned from mental health enhancement and suicide prevention activities in the Veterans Health Administration. *American Journal of Public Health, 102*(Supplement 1), S14-S16.

Lerche, V., Voss, A., & Nagler, M. (2017, Apr). How many trials are required for parameter estimation in diffusion modeling? A comparison of different optimization criteria. *Behavior Research Methods, 49*(2), 513-537.

Matarazzo, B. B., Clemans, T. A., Silverman, M. M., & al., e. (2013). The self-directed violence classification system and the Columbia Classification Algorithm for suicide assessment: A crosswalk. *Suicide and Life-Threatening Behavior, 43*(3), 235-249.

Posner, K., Brown, G., Stanley, B., Brent, D. A., Yershova, K. V., Oquendo, M. A., . . . Mann, J. J. (2011). The Columbia-Suicide Severity Rating Scale (C-SSRS): Initial validity and internal consistency findings from three multi-site studies with adolescents and adults. *American Journal of Psychiatry, 168*(12), 1266-1277.

R Core Team. (2017). *R: A language and environment for statistical computing.* R Foundation for Statistical Computing. <https://www.R-project.org/>

Ratcliff, R. (1978). A theory of memory retrieval. *Psychological Review, 85*(2), 59-108.

Ratcliff, R., & Childers, R. (2015). Individual Differences and Fitting Methods for the Two-Choice Diffusion Model of Decision Making. *Decision (Washington, DC), 2015*, 10.1037/dec0000030.

Ratcliff, R., & McKoon, G. (2008). The diffusion decision model: Theory and data for two-choice decision tasks. *Neural Computation, 20*, 873-922.

Ratcliff, R., Smith, P. L., Brown, S. D., & McKoon, G. (2016). Diffusion decision model: Current issues and history. *Trends in Cognitive Sciences, 20*(4), 260-281.

Sheehan, D. V., Lecrubier, Y., Sheehan, K. H., Amorim, P., Janavs, J., Weiller, E., . . . Dunbar, G. C. (1998). Neuropsychiatric Interview (M.I.N.I.): The development and validation of a structured diagnostic psychiatric interview for DSM-IV and ICD-10. *Journal of Clinical Psychiatry, 59 Suppl 20*, 22-33.

Stanley, B., & Brown, G. K. (2012). Safety planning intervention: A brief intervention to mitigate suicide risk. *Cognitive and Behavioral Practice, 19*(2), 256-264.

Turner, B. M., Sederberg, P. B., Brown, S. D., & Steyvers, M. (2013). A method for efficiently sampling from distributions with uncorrelated dimensions. *Psychological Methods, 18*, 368-384.

White, C. N., Servant, M., & Logan, G. D. (2018). Testing the validity of conflict drift-diffusion models for use in estimating cognitive processes: A parameter-recovery study. *Psychonomic Bulletin and Review, 25*(1), 286-301.
